# Simulating Cancer Recurrence Patterns From Post-Treatment Viable Tumor Burden Distributions

**DOI:** 10.1200/CCI-25-00072

**Published:** 2026-05-08

**Authors:** Mohammad U. Zahid, Joseph D. Butner, David M. Swanson, David A. Hormuth, Heiko Enderling

**Affiliations:** ^1^ Department of Radiation Oncology, The University of Texas MD Anderson Cancer Center, Houston, TX; ^2^ Department of Biostatistics, The University of Texas MD Anderson Cancer Center, Houston, TX; ^3^ Oden Institute for Computational Engineering and Sciences, The University of Texas at Austin, Austin, TX; ^4^ Livestrong Cancer Institutes, The University of Texas at Austin, Austin, TX; ^5^ Institute for Data Science in Oncology, The University of Texas MD Anderson Cancer Center, Houston, TX

## Abstract

**PURPOSE:**

Ordinary differential equation mathematical models of tumor volume dynamics can accurately describe tumor growth and treatment response. Here, we extend such continuous models to also simulate outcomes. We conceptualize post-treatment viable tumor burden distributions across a treatment population and a novel model of tumor regrowth that can simulate population-level recurrence patterns.

**METHODS:**

We use a mathematical model of tumor regrowth dynamics that is attenuated by a minimum viable tumor burden threshold (ε_V_) below which the tumor will be cured. Tumor regrowth is simulated until the tumor burden exceeds a detection threshold (ω_d_), which allows for the modeling of Kaplan-Meier curves.

**RESULTS:**

We then explore the effect of the different model parameters and growth laws on the shapes of simulated Kaplan-Meier curves and demonstrate how this model can be used to further our understanding of clinical trial results. We also present qualitative fitting of this model to real-world recurrence data from a clinical trial comparing different radiation therapy protocols in head and neck cancer (RTOG 9003).

**CONCLUSION:**

The theoretical framework described in this brief report provides a means to connect models of tumor dynamics to recurrence patterns. We foresee that it will also provide a new methodology for interpreting the shapes of Kaplan-Meier curves and provide insights as to why particular clinical trials failed and guide how to redesign them for success.

## INTRODUCTION

There has been an explosion in predictive models for oncology, ranging from mechanistic mathematical models to data-driven machine learning and AI models.^1^ While some machine learning models have been successfully deployed for predicting cancer recurrence, they often fail to provide mechanistic understanding of what underlies or drives the recurrence of disease.^2-7^ Similarly, cure fraction models explicitly fit the fraction of patients who will eventually be cured by a treatment intervention without providing a mechanistic explanation.^8-10^ Ordinary and partial differential equation models successfully simulate changes in tumor volume in response to treatment.^11-19^ To simulate outcomes, recurrence could be defined as the simulated post-treatment tumor burden exceeding some defined threshold.^20-22^ To simulate cure, some mechanistic models set the tumor burden to zero below the threshold of one tumor cell,^23^ whereas others have used hybrid deterministic-stochastic approaches to capture stochastic extinction events that occur at lower cell numbers.^24^ Here, we introduce a novel continuous mechanistic ordinary differential equation model that simulates disease recurrence and durable response and cure. We present a framework for connecting distributions of post-treatment residual viable tumor burden to recurrence curves using a mathematical model of tumor clearance as well as regrowth and recurrence.

## METHODS

**FIG 1.**
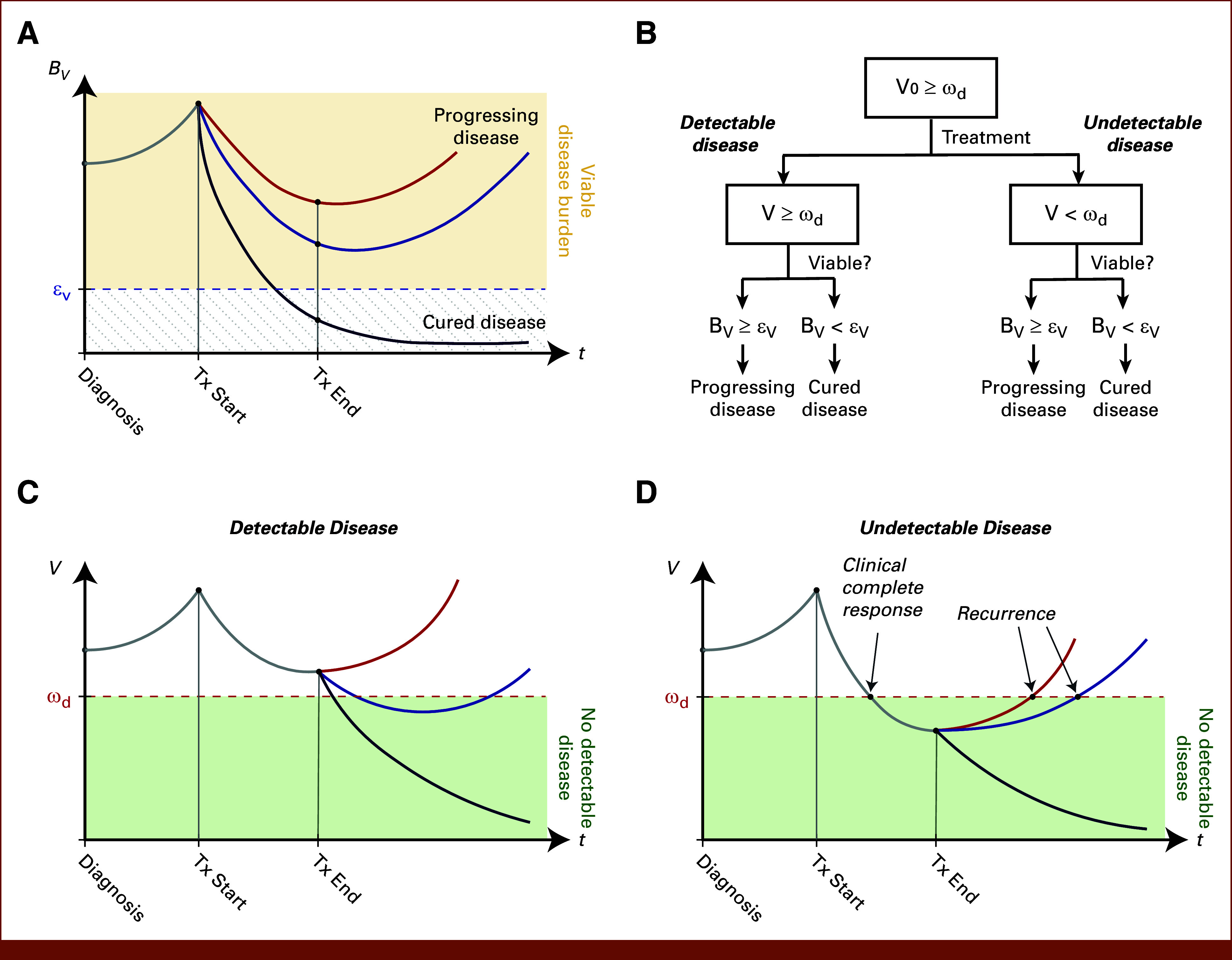
Conceptual depictions of how viable tumor burden ($B_{V}$) and tumor volume (V) change over time. (A) Viable disease burden ($B_{V}$) increases from diagnosis to the start of treatment and decreases for all patients from the start of treatment until the end of treatment. The difference between eventual progression and cure may defined by whether $B_{V}$ is above or below the minimum viability threshold $\varepsilon_{V}$ at the end of treatment. (B) Flowchart depicting the possible paths during the treatment journey of a patient with cancer. Before treatment, all patients presumably have detectable disease (ie, $V_{0}\;>\;\omega_{d}$). After treatment, patients might have detectable or undetectable disease, and in both these cases, the viable disease burden will either be above (leading to disease progression) or below (leading to cure) the minimum viability threshold $\varepsilon_{V}$. (C) Detectable disease at the end of treatment ($V\;>\;\omega_{d}$), which can lead to different trajectories that result in progression ($B_{V}>\varepsilon_{V}$) or cure ($B_{V}<\varepsilon_{V}$). (D) Undetectable disease at the end of treatment ($V\leq \omega_{d}$), which can also lead to different trajectories that result in progression ($B_{V}>\varepsilon_{V}$) or cure ($B_{V}<\varepsilon_{V}$), despite complete response at the end of treatment.

We assume that at the time of diagnosis, a patient has detectable tumor volume that has some proportion of viable disease burden ($B_{V}$) that is continuing to grow until the start of therapy. We further assume that during the course of treatment, $B_{V}$ will decrease, but after the cessation of treatment, some patients might have disease progression, whereas other patients' tumors may be cured (Fig 1A). To explain why some patients have tumors that regrow and ultimately recur, while some do not, we propose a minimum viable tumor burden, $\varepsilon_{V}$, that is necessary for a tumor to survive in the post-treatment tumor environment and to regrow to a detectable size, comparable with the Allee effect in population ecology.^25-28^ We define a threshold for the minimum viable residual tumor burden ($\varepsilon_{V}$) as the smallest tumor that could regrow to clinical detection after therapy. With this minimum viability threshold, it may be possible to explain the differences in patient outcome (ie, recurrence v nonrecurrence, early *v* late recurrence) using a mathematical model of post-treatment tumor regrowth. This threshold is defined to be non-negative, meaning that it can take on any value greater than or equal to zero, including even a single cancer cell.

The amount of viable disease burden ($B_{V}$) is a subset of the post-treatment tumor volume, which may be significantly less than the total tumor volume. After the cessation of treatment, a patient might have a detectable tumor volume or they might have no detectable disease. To describe these cases, we introduce a detection threshold, $\omega_{d}$, above which the tumor can be clinically detected. In the case of both detectable ($B_{V}\geq \omega_{d}$) and undetectable ($B_{V}<\omega_{d}$) diseases, $B_{V}$ may be above or below the minimum viability threshold $\varepsilon_{v}$ (Fig 1B). Thus, even detectable disease can result in either progression or cure (Fig 1C). Undetectable disease at the end of treatment, which can be classified as complete clinical response, might also have eventual disease recurrence, despite there being no detectable disease at the end of treatment (Fig 1D). This motivates modeling viable disease burden dynamics.

**FIG 2.**
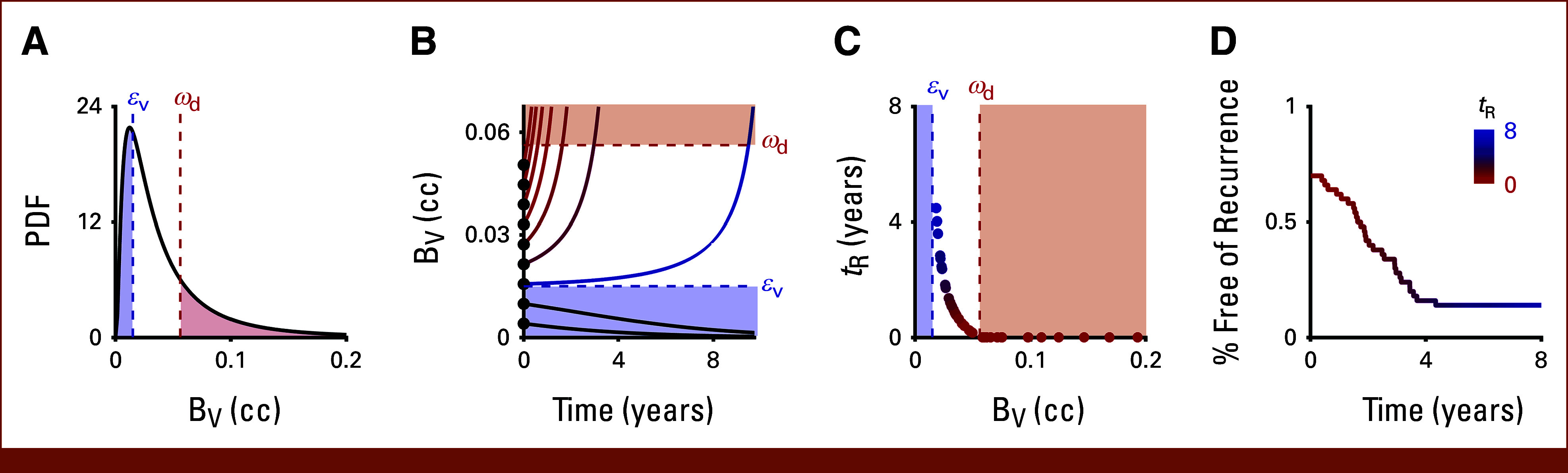
Going from simulated viable tumor burden ($B_{V}$) to a recurrence curve: (A) Lognormal probability distribution (mean = 0.05 cc, σ = 0.06) of $B_{V}$, with minimum viable tumor volume threshold ($\varepsilon_{v}$) and detection threshold ($\omega_{d}$) indicated in blue and red, respectively. (B) Example simulations of tumor regrowth/decay (with λ = 0.0008 d^−1^) for n = 9 equally spaced virtual patients sampled from the original $B_{V}$ distribution (A), where black dots indicate the initial sampled tumor volume, and the volume where the regrowing tumor becomes detectable (crosses the $\omega_{d}$ threshold) is indicated by the red dashed line. Note that the trajectory with $B_{V}<\varepsilon_{V}$ decays to zero. (C) Scatter plot of time to recurrence as a function of the residual viable tumor volume for n = 50 virtual patients, where the color of the points indicates the time to recurrence ($t_{R}$) in years. (D) Recurrence curve for the same n = 50 virtual patients from (C).

We assume that, after a given cancer treatment, individual patients might have different amounts of residual viable tumor burden, $B_{V}$, based on a combination of factors, such as differences in pretreatment disease burden and response to therapy (Fig 2A).^29-31^ The post-treatment viable tumor burden dynamics are described by

(1) $$\frac{dB_{V}}{dt}=f\left(V\right)\cdot \left(\frac{B_{V}}{\varepsilon_{V}}-1\right),$$

where $\frac{dB_{V}}{dt}$ is the change in viable tumor burden, t is the time from the end of treatment, f(V) is the tumor growth law that describes how untreated tumor volume changes over time,^32^ and $\varepsilon_{v}$ is the previously defined minimum viable residual tumor burden. In this formulation, the tumor volume $B_{V}\left(t\right)$ goes to zero as t→∞ if and only if $B_{V}<\varepsilon_{V}$ and grows if and only if $B_{V}>\varepsilon_{V}$ (Fig 2B). For initial demonstration purposes (and without loss of generality), we use exponential growth as the tumor growth law, that is, f(V)=λV, where λ [time^–1^] is the intrinsic tumor growth rate. Exponential growth as the intrinsic growth law, modified by the minimum viability term, is intuitive from the general assumption that the viable residual tumor burden is significantly lower than any putative untreated carrying capacity-related growth rate modulators.^14,32-34^ The effect of other functional forms of f(V) on post-treatment tumor dynamics and the shape of the associated recurrence curves will be analyzed further below. Of note, the net change in tumor volume in the functional form of Equation 1 is not identical to f(V), that is, setting f(V)=λV as exponential growth does not imply that $\frac{dB_{V}}{dt}$ follows exponential growth dynamics (Fig 2B). For simplicity, and in line with previous analyses of clinical data, we assume that all patients have the same tumor regrowth rate, λ.^12,35-37^ However, with sufficient clinical response and outcome data, it should be possible to test this assumption and, if necessary, estimate patient-specific values for λ as a function of various clinical covariates.

To model recurrence as a function of time, we formally define the detection threshold, $\omega_{d}$, above which the tumor can be clinically detected as follows:

(2) $$R_{i}\left(t\right)=\left\{ \begin{array}{c} \;0,\;\;\;B_{V}\left(t\right)<\omega_{d}\\ \;1,\;\;\;B_{V}\left(t\right)\geq \omega_{d} \end{array}\right.,$$

where $R_{i}\left(t\right)$ is the recurrence as a function of time for patient i. Here, R=0 when there is no recurrence and R=1 when recurrence occurs. Thus, for each individual patient with $B_{V}>\varepsilon_{V}$, a recurrence time can be calculated based on their value of $B_{V}$ (Fig 2C). These individual patient recurrence curves can then be averaged over time to construct the full recurrence curve (Fig 2D) with

$$R\left(t\right)=\frac{1}{n_{pts}}\overset{n_{pts}}{\underset{i=1}{\sum }}R_{i}\left(t\right),$$ (3)

where $n_{pts}$ is the total number of patients and R(t) is the recurrence rate for the whole cohort as a function of time. To represent this as a Kaplan-Meier curve, we plot 1−R(t), which corresponds to the percentage of the population free from recurrence as a function of time. Of note, this method is only appropriate for simulating recurrence from residual viable tumor burden distributions and not appropriate for modeling overall survival.

## RESULTS

### Effects of Model Parameters

**FIG 3.**
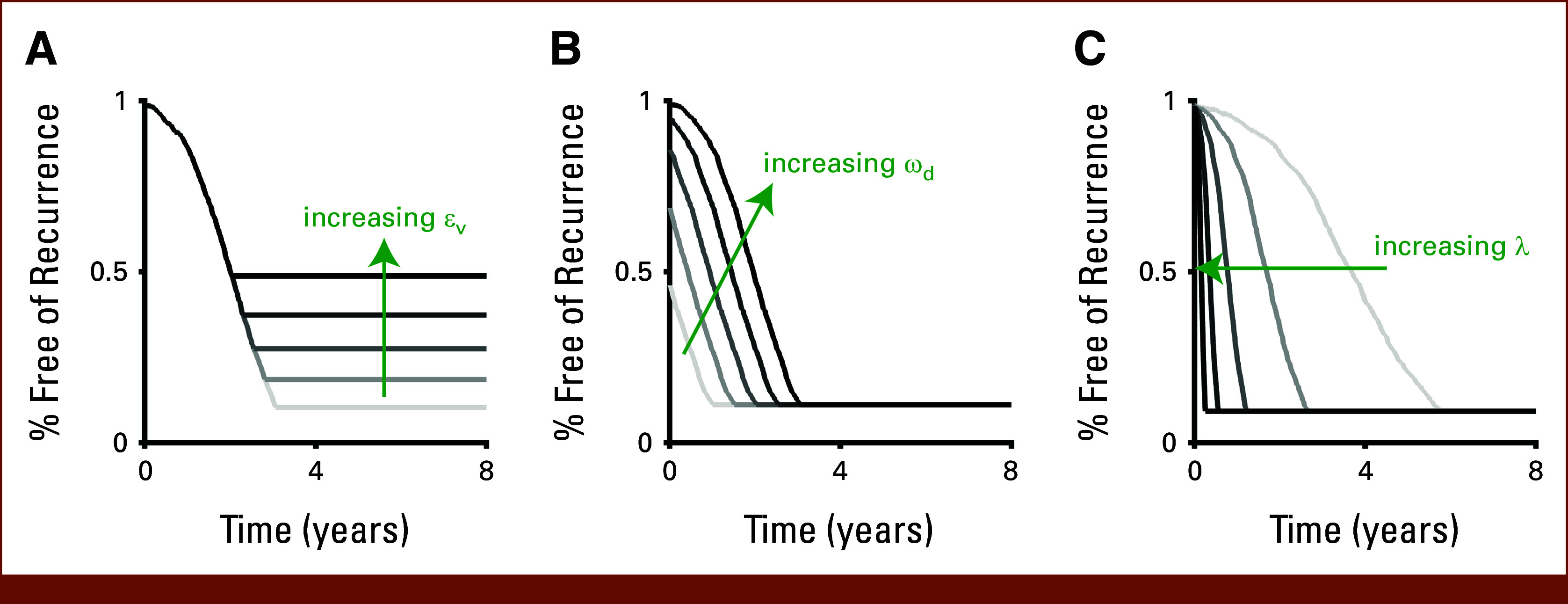
Effect of model parameters on the recurrence curve shape. Recurrence curves generated using n = 1,000 virtual patients. (A) The effect of increasing $\varepsilon_{V}$ on the range of (0.010, 0.018) cc, (B) $\omega_{d}$ on the range of (0.018, 0.056) cc, and (C) λ on the range of (0.0008, 0.018) d^−1^. All parameters were uniformly sampled from the stated parameter ranges. Baseline parameter values used for these simulations are $\varepsilon_{V}$ = 0.01 cc, $\omega_{d}$ = 0.056 cc, and λ = 0.0015 d^−1^.

We examine the effect of each of the model parameters on different features of the survival curves. The viability threshold $\varepsilon_{V}$ controls the minimum value that the curve will asymptote to (Fig 3A). This is analogous to how a cure fraction is deployed in the eponymous cure models.^8-10,38^ The detection threshold $\omega_{d}$ controls the y-intercept of the curve (Fig 3B). This indicates the proportion of the $B_{V}$ distributions that is already above the detection threshold at t = 0. The tumor regrowth rate λ controls the rate of the drop-off between the y-intercept and the time at which the curve approaches the floor defined by $\varepsilon_{V}$ (Fig 3C).

### Simulating a Clinical Trial Scenario

**FIG 4.**
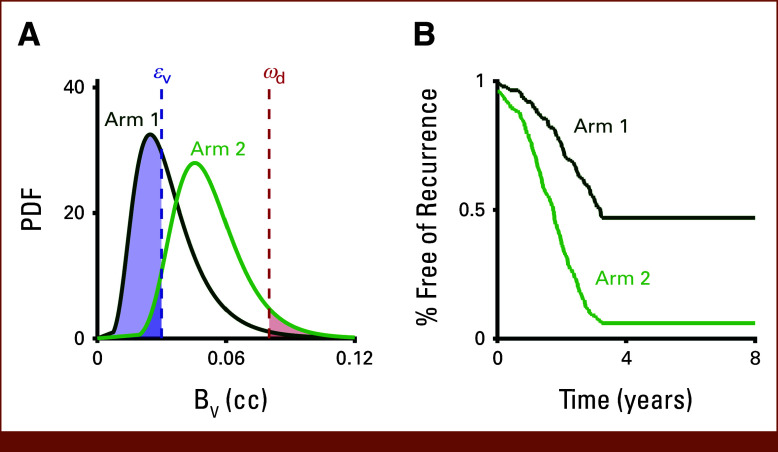
Example scenario where the two arms of a trial may result in different $B_{V}$ distributions which lead to different survival curves. (A) Two simulated lognormal $B_{V}$ distributions with minimum viable tumor burden threshold ($\varepsilon_{v}$) and detection threshold ($\omega_{d}$) indicated in blue and red respectively. (B) The corresponding simulated recurrence curves for n = 200 patients sampled for each arm.

We simulate two $B_{V}$ distributions for patients in two different arms of a putative clinical trial. The $B_{V}$ distribution for Arm 2 has a slightly higher mode and a long tail that extends past $\omega_{d}$ (Fig 4A). Assuming that the two patient populations had comparable distributions of tumor burden pretreatment, the more effective treatment has a lower average value of $B_{V}$ (Arm 1, mean = 0.03 cc) relative to the less effective treatment (Arm 2, mean = 0.05 cc). With all other parameters held constant between the two arms, the curves were calculated for n = 200 virtual patients sampled from each arm (Fig 4B). The lower average value of the $B_{V}$ distribution of Arm one results in later average recurrence. In addition, the larger proportion of the $B_{V}$ distribution below $\varepsilon_{V}$ in Arm 1 (49.4% in Arm 1 *v* 4.6% in Arm 2) yields the curve to asymptote to a higher value (0.49 in Arm 1 *v* 0.045 in Arm 2). Interestingly, the changes in concavity of the curves in Fig 4B map to changes according to the sign of the first derivative of corresponding $B_{V}$ probability distribution function curves in Fig 4A.

### Effects of Tumor Growth Laws

**FIG 5.**
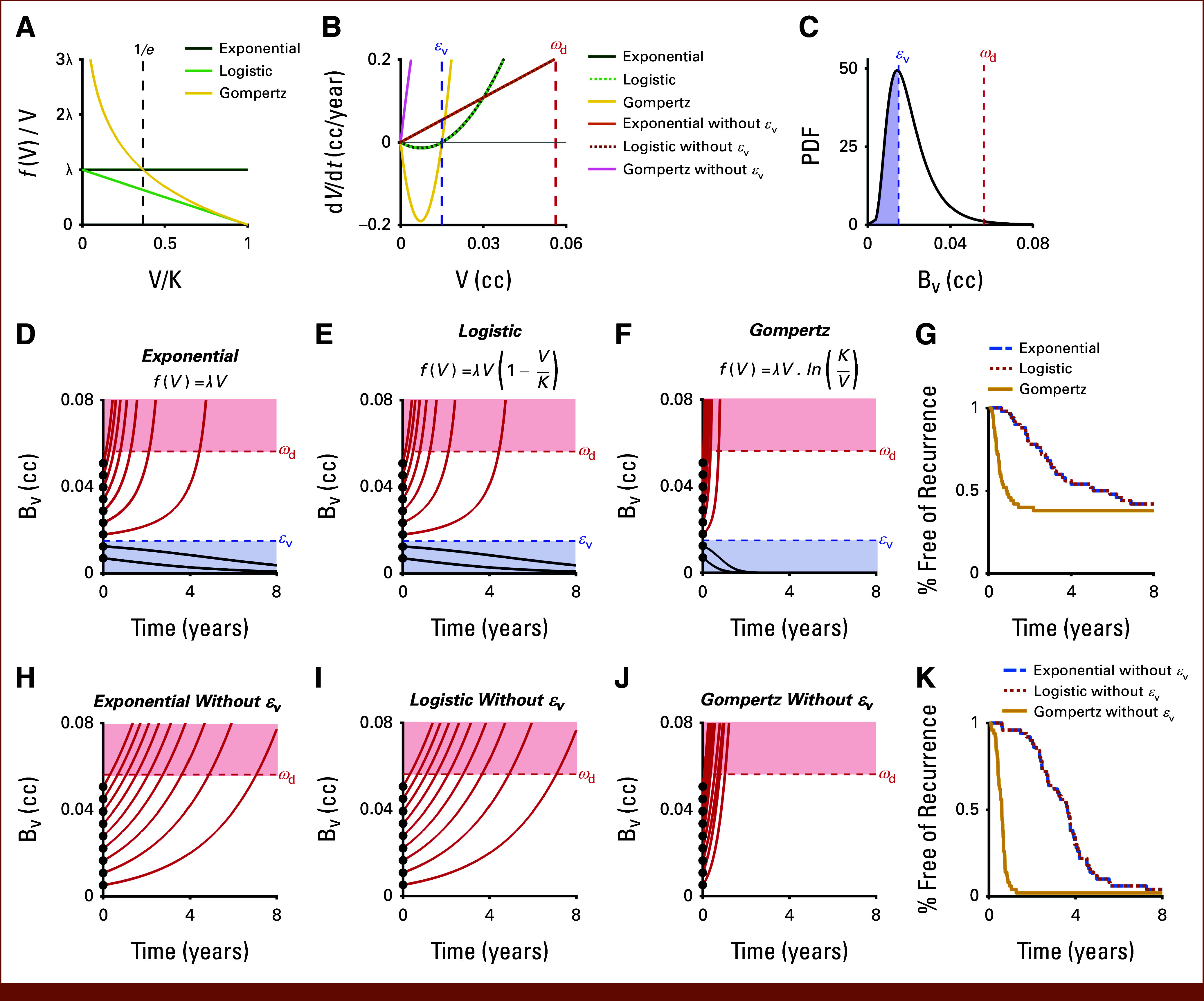
Comparing the effects of different tumor growth laws. (A) Plots of relative values of the different tumor growth laws, that is, f(V)/V, against V/K demonstrating how logistic growth exhibits subexponential growth that diminishes steadily, whereas Gompertz growth has superexponential growth at values of V/K < 1/e. (B) Comparisons of net growth, that is, dV/dt, for all the tumor growth laws with and without the minimum viability term. Note that dV/dt is only ever negative for the models with the minimum viability term and then only for $V<\;\varepsilon_{V}$. (C) A fixed lognormal $B_{V}$ distribution (mean = 0.02 cc, σ = 0.5) was used for the growth law comparison simulations with $\varepsilon_{V}$ = 0.015 cc and $\omega_{d}$ = 0.056 cc. Example simulations of tumor regrowth/decay with $\varepsilon_{V}$ using (D) modified exponential, (E) logistic, and (F) Gompertz growth laws with λ = 0.0009 day^−1^. The same n = 9 equally spaced virtual patients sampled from the original $B_{V}$ distribution (C) are used for each growth law. The equations for each growth law are indicated in their respective panels. (G) Recurrence curves for n = 50 virtual patients using the three different tumor growth laws. Example simulations of tumor regrowth/decay without $\varepsilon_{V}$ using (H) exponential, (I) logistic, and (J) Gompertz growth laws with λ = 0.0009 d^−1^. The same n = 9 equally spaced virtual patients sampled from the original $B_{V}$ distribution (C) are used for each growth law. The equations for each growth law are indicated in their respective panels. (K) Recurrence curves for n = 50 virtual patients using the three different tumor growth laws.

As discussed above, the choice of exponential growth for the tumor growth law in f(V) in Equation 1 yields characteristic recurrence curves. Next, we examine the impact of other classic growth laws^32,33,39,40^ for f(V) in Equation 1: logistic growth with $f\left(V\right)=\lambda V\left(1-\frac{V}{K}\right)$^41,42^ and Gompertzian growth with $f\left(V\right)=\lambda V\cdot \ln\left(\frac{K}{V}\right)$.^43,44^ Both logistic and Gompertzian growth include the carrying capacity, K*,* as the maximum tumor volume that can be supported by the local tissue environment. The effect of the logistic and Gompertz function on the relative value of f(V) is shown in Figure 5A, with a linear decline in logistic growth as V approaches K (and $\frac{V}{K}$ approaches 1), superexponential growth in Gompertz growth as $\frac{V}{K}<\frac{1}{e}$, and rapid deceleration of growth as $\frac{V}{K}$ approaches 1 beyond $\frac{1}{e}$. As mentioned previously, without the minimum viability modification that our model uses, Gompertz growth yields superexponential net growth rates in our volume range of interest (ie, $V\;<\;\omega_{d}$), and logistic growth yields net growth rates indistinguishable from exponential growth (Fig 5B). In addition, without the minimum viability modification, all three growth models always have dV/dt >0. On the other hand, with the minimum viability modification, all three models have dV/dt<0 for the range of $V<\varepsilon_{V}$, with the exponential and logistic growth models having indistinguishable net growth rates for $V\;<\;\omega_{d}$. As noted earlier, these two models do not, however, exhibit standard exponential growth patterns—rather, they have a subexponential growth phase followed by a superexponential growth phase at larger tumor volumes. This is the same for Gompertzian growth, but the subexponential phase is much shorter (Fig 5B).

To compare the effect of exponential growth with logistic and Gompertz growth laws in Equation 1 on tumor recurrence patterns, we sample from the same $V_{R}$ distribution with fixed value for $\omega_{d}$ and $\varepsilon_{v}$ (Fig 5C) and then simulate tumor volume dynamics (Figs 5D-5F) and recurrence-free survival patterns (Fig 5G). For logistic and Gompertzian growth, each virtual patient had a patient-specific value for K sampled from a lognormal distribution with values three orders of magnitude higher than the $B_{V}$ distribution (mean = 13.8 cc, std. dev. = 0.5). The recurrence curve generated using logistic growth is indistinguishable from the curve made using exponential growth (Fig 5G) as $B_{V}$ and $\omega_{d}$ are orders of magnitude smaller than the values of K (ie, $\frac{B_{V}}{K}=0.005$ when $B_{V}=\omega_{d}$), in which case logistic growth approximates exponential growth ($\lambda B_{V}$ *v* [$0.996\lambda *B_{V}$]). As described above, Gompertzian growth yields superexponential growth at low values (Fig 5A), with $\ln\left(\frac{K}{B_{V}}\right)=5.44$ when $B_{V}=\omega_{d}$. Compared with exponential growth, this yields a 5.44 times faster growth rate at $V_{\omega_{d}}$ and thus rapid recurrence patterns (Fig 5B) and lower recurrence curves (Fig 5G). Even with a fixed growth rate, λ, different growth laws f(V) in Equation 1 lead not only to different rates of recurrence but also to different shapes of the recurrence curves (Fig 5G). Recurrence curves from Gompertz growth demonstrate an inverted curvature compared with exponential growth. This demonstrates the need to calibrate the demonstrated methodology and its underlying growth laws to the clinically observed recurrence patterns of specific cancer types. Furthermore, we repeated these simulations without the minimum viability threshold, but with otherwise identical parameters, for all three growth laws (Figs 5H-5K). All three growth laws show that all simulations eventually exceed $\omega_{d}$ without the minimum viability threshold (Figs 5H-5J) and that the corresponding recurrence curves all eventually go to zero (Fig 5K).

### Qualitative Fitting to Real-World Recurrence Data

To demonstrate the utility in explaining real clinical recurrence patterns, we fit the tumor regrowth/recurrence model to published locoregional control (LRC) data from RTOG 9003, a phase III randomized study comparing different radiation therapy (RT) fractionation schedules in patients with head and neck squamous cell (HNSCC).^45,46^ Specifically, we used the LRC data from standard fractionated RT (ie, 2 Gy daily fractions) and hyperfractionated RT (ie, 1.2 Gy twice daily fractions).

**FIG 6.**
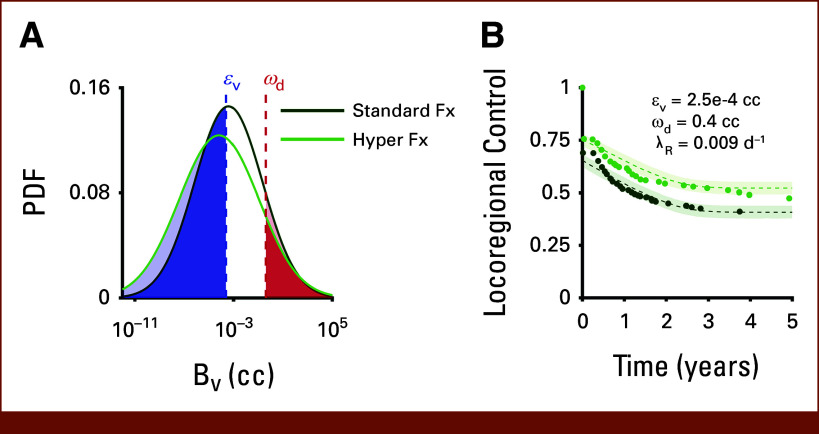
Example fitting of survival data from RTOG 9003. (A) $B_{V}$ distributions were estimated for both arms by simulating standard and hyperfractionated radiation therapy in accordance with the treatment protocols from RTOG 9003. (B) Plots of mean locoregional control curve fits (dashed lines) with 95% confidence internals for both treatment arms from 100 simulations of sampling $B_{V}$ values from the distributions in (A) and using global values of $\varepsilon_{V}=2.5\times 10^{-4}$ cc, $\omega_{d}=0.4$ cc, and $\lambda_{R}=0.009$ d^−1^. Filled circles are abstracted locoregional control data from RTOG 9003.

First, we simulated $V_{R}$ distributions for both treatment arms (Fig 6A). This was performed by assuming a lognormal distribution of pretreatment tumor volume, $V_{0}∼lognormal\left(\mu =2.8,\sigma =0.82\right)$, where μ and σ are the location and scale parameters the define the lognormal distribution, based on previously published HNSCC data from comparable cohorts. Next, we estimated $V_{R}$ distributions by simulating the full RT course according to our previously published tumor volume dynamic models.^11,47,48^ This was done by sampling n = 265 virtual patients from the $V_{0}$ distribution, simulating the both standard and hyperfractionated RT for each virtual patient and then fitting lognormal distributions to the calculated final tumor volumes. This yielded $B_{V}∼lognormal\left(\mu =-8.0,\sigma =6.8\right)$ for the standard fractionation arm and $B_{V}∼lognormal\left(\mu =-10.0,\sigma =7.6\right)$ for the hyperfractionation arm.

We then calibrated the tumor regrowth/recurrence model parameters ($\lambda ,\omega_{d},\varepsilon_{V}$) to fit the LRC curves from RTOG 9003 (Fig 6B). This was done by sampling from both $B_{V}$ distributions, running the regrowth/recurrence model 100 times, and finding model parameters that minimized the error to the LRC curves. This yielded values of $\lambda =0.005\;days^{-1}$, $\omega_{d}=0.4\;cc$, and $\varepsilon_{V}=2.5\times 10^{-4}cc$. The differences between the two treatment arms can potentially be explained by the differences between the two $B_{V}$ distributions, where the higher asymptote of the hyperfractionation LRC curve corresponds to a larger proportion of the $V_{R}$ distribution being below the $\varepsilon_{V}$ threshold.

## DISCUSSION

In conclusion, herein, we presented a mathematical model to simulate survival curves of disease recurrence as a function of a post-treatment residual viable tumor burden distributions, the tumor regrowth rate, a minimum tumor viability threshold, and a recurrence detection threshold. This framework allows us to connect mechanistic tumor response modeling with mechanistic outcome modeling—a heretofore limited capability of differential equation models in oncology. In its current form, the model is configured to simulate regrowth after cytoreductive therapies (eg, surgery, radiotherapy, some chemotherapies, etc). It is straightforward, however, to leverage this model for other mechanisms of action. For example, one could simulate a treatment that alters tumor proliferation by using different growth rate values during and after therapy or, when comparing different drugs in different arms of a clinical trial, different λ values for the patients in the different treatment arms.

Conceptualizing survival curve features through mechanistic mathematical models offers a novel quantitative analysis of survival data and allows for a better understanding of how interventions in clinical trials are performed across a patient population. The herein presented framework is simulating outcome statistics from distributions of residual viable tumor burden. It is conceivable that this framework could be used in reverse to infer response to different therapies from outcome statistics of multiple clinical trial arms. In this case, precise parameter identification may be immaterial as the differences between the inferred $V_{R}$ distributions and estimated parameter values may be sufficient for understanding the differences between trial arms. If this approach ultimately enables a quantitative analysis of the time-resolved survival data beyond whether a particular treatment yields survival benefit over another treatment, then it may be possible to deepen our understanding of clinical trial results. For instance, this approach may provide insights as to why particular clinical trials failed and guide how to redesign them for success. In addition, one could perform in silico clinical trials with different virtual patient numbers in the different treatment arms to simulate required patient numbers to achieve statistical significance for desired outcome statistics.

Parameter identifiability (ie, being able to correctly identify unique parameter values) may be a limitation for the inverse problem of estimating $B_{V}$ distributions and model parameters from retrospective clinical trial survival data. These limitations could be addressed through parameter reduction methods by which a subset of parameters is fixed before fitting the model to survival data.^1^ Another model assumption is that the parameters are uniform across the patient population. This assumption is made in the interest of developing the most parsimonious model given the available data. However, in the case where the model does not sufficiently describe the data (ie, unable to fit the data without patient-specific parameter values), then additional degrees of freedom can be introduced by treating the parameters which are likely to have interpatient heterogeneity, such as $\varepsilon_{V}$ and λ (as opposed to $\omega_{d}$, which is more likely to be a function of instrumentation or biological limits), as distributed parameters with patient-specific values.

In addition, we only demonstrated examples of unimodal $B_{V}$ distributions, which assume unimodal distributions of both pretreatment tumor volumes and treatment effects. However, these assumptions can be relaxed given appropriate clinical data by fitting multimodal $B_{V}$ distributions to represent potential subpopulations with variable characteristics.

To systematically fit this model to real-world recurrence data, several additional factors will need to be considered. In its current form, the model assumes perfect information and continuous monitoring, such that recurrence is determined as soon as the regrowing tumor is detectable. For fitting to clinical data, appropriate surveillance schedules will need to be accounted for, as well as left and right censoring for various clinical events.

As presented here, the model is designed to simulate disease recurrence, which we have defined as when tumor regrows beyond some detection threshold, $\omega_{d}$. This means that the model predictions will also be sensitive to the factors that influence detection of disease, such as the imaging modality used for detection. For example, a more sensitive modality, such as PET, might have a lower value for the detection threshold, compared with less sensitive modality, such as CT. In addition, it would be straightforward to adapt the model to simulate disease progression as assessed by RECIST.^49^ This could be accomplished by normalizing the residual viable tumor volume to the baseline tumor volume or volume nadir for each patient and using the RECIST progression threshold in the place of $\omega_{d}$.

Beyond demonstration herein, it is conceivable that the presented approach will further enable more accurate calibration of treatment response dynamic differential equation models. Longitudinal clinical measurements before and during therapy are limited for individual patients, which severely constrain mathematical model parameter identifiability and thus model complexity. Time-resolved outcome data, such as cure, progression, and recurrence, will allow us to further constraint and calibrate parameters in on-treatment response models as minimal residual viable burden distributions can now also be used for calibration. Ultimately, this presented theoretical framework will not only serve to connect models of tumor dynamics with survival curves by providing a way to calibrate these models with a readily available data type but also provide a new methodology for interpreting the shapes of recurrence curves as a function of tumor regrowth and the distribution of viable residual tumor burden in a patient population.

## Data Availability

Code and materials used for this study are available upon reasonable request.
